# Golden bananas in the field: elevated fruit pro‐vitamin A from the expression of a single banana transgene

**DOI:** 10.1111/pbi.12650

**Published:** 2016-12-20

**Authors:** Jean‐Yves Paul, Harjeet Khanna, Jennifer Kleidon, Phuong Hoang, Jason Geijskes, Jeff Daniells, Ella Zaplin, Yvonne Rosenberg, Anthony James, Bulukani Mlalazi, Pradeep Deo, Geofrey Arinaitwe, Priver Namanya, Douglas Becker, James Tindamanyire, Wilberforce Tushemereirwe, Robert Harding, James Dale

**Affiliations:** ^1^ Centre for Tropical Crops and Biocommodities Queensland University of Technology Brisbane Qld Australia; ^2^ Agri‐Science Queensland Department of Agriculture and Fisheries South Johnstone Qld Australia; ^3^ PlantVax Inc Rockville MD USA; ^4^ National Agricultural Research Laboratories National Agricultural Research Organization Kampala Uganda; ^5^ Present address: Sugar Research Australia Brisbane Qld Australia; ^6^ Present address: Syngenta Asia Pacific Singapore Singapore; ^7^ Present address: Charles Sturt University Wagga Wagga NSW Australia

**Keywords:** Vitamin A deficiency, Uganda, pro‐vitamin A, staple food crop, banana, biofortification, genetic modification

## Abstract

Vitamin A deficiency remains one of the world's major public health problems despite food fortification and supplements strategies. Biofortification of staple crops with enhanced levels of pro‐vitamin A (PVA) offers a sustainable alternative strategy to both food fortification and supplementation. As a proof of concept, PVA‐biofortified transgenic Cavendish bananas were generated and field trialed in Australia with the aim of achieving a target level of 20 μg/g of dry weight (dw) β‐carotene equivalent (β‐CE) in the fruit. Expression of a Fe'i banana‐derived phytoene synthase 2a (*MtPsy2a*) gene resulted in the generation of lines with PVA levels exceeding the target level with one line reaching 55 μg/g dw β‐CE **.** Expression of the maize phytoene synthase 1 (*ZmPsy1*) gene, used to develop ‘Golden Rice 2’, also resulted in increased fruit PVA levels although many lines displayed undesirable phenotypes. Constitutive expression of either transgene with the maize polyubiquitin promoter increased PVA accumulation from the earliest stage of fruit development. In contrast, PVA accumulation was restricted to the late stages of fruit development when either the banana 1‐aminocyclopropane‐1‐carboxylate oxidase or the expansin 1 promoters were used to drive the same transgenes. Wild‐type plants with the longest fruit development time had also the highest fruit PVA concentrations. The results from this study suggest that early activation of the rate‐limiting enzyme in the carotenoid biosynthetic pathway and extended fruit maturation time are essential factors to achieve optimal PVA concentrations in banana fruit.

## Introduction

Micronutrient deficiency, often referred to as hidden hunger, occurs when intake and absorption of vitamins and minerals are too low to sustain good health and development. The World Health Organization (WHO) estimates that 190 million pre‐school children are deficient in one of the major micronutrients, vitamin A. Vitamin A deficiency (VAD) alone is responsible for almost 6% of child deaths under the age of 60 months in Africa and 8% in South‐East Asia (WHO, 2011). For the vast majority of these children, VAD is almost exclusively the result of inadequate intake of dietary vitamin A or pro‐vitamin A (PVA) although exacerbated by other health conditions. Similar levels of VAD are also evident in women of childbearing age in these same regions (WHO, 2011). These levels of VAD continue despite the implementation over many years of extensive alleviating strategies such as supplements and food fortification. These strategies have been demonstrably successful, but there remain persistently high and unacceptable levels of VAD particularly in sub‐Saharan Africa and south Asia (Stevens *et al*., 2015).

In an effort to significantly reduce VAD in these regions, strategies aimed at increasing the dietary intake of particularly α‐ and β‐carotene together as PVA are being developed or implemented. These include programmes to encourage growing and consuming staple foods with high levels of PVA. In some instances, such foods or crops with the desired agronomic and consumer traits are already available and can therefore be easily deployed (HarvestPlus, 2012). However, the majority of accepted cultivars and landraces of staple crops are low in micronutrients such as PVA and iron, and therefore, it is necessary to develop new varieties with enhanced levels of these micronutrients. This can be achieved either through conventional breeding or by genetic modification where the traits are not available within the accessible germplasm or cannot be easily introgressed into acceptable cultivars. These two approaches are known as biofortification.

The best‐known example of biofortification by genetic modification, and the most advanced in terms of development, is ‘Golden Rice’. In Ye *et al*., 2000 and colleagues reported the generation of transgenic rice expressing the daffodil phytoene synthase (*Psy*) gene under the control of an endosperm‐specific rice glutelin promoter together with the bacterial (*Pantoea ananatis* formerly known as *Erwinia uredovora*) phytoene desaturase (*CrtI*) gene under the control of the constitutive CaMV 35S promoter. The endosperm of selected lines was yellow, and one heterozygous line contained 1.6 μg/g dry weight (dw) total carotenoids. Paine *et al*. (2005) subsequently reported the development of the second generation of ‘Golden Rice’, which was engineered with a maize (*Zea mays*) *Psy* gene and the *CrtI* gene under the control of the glutelin promoter. One Golden Rice 2 elite event had a 23‐fold increase in total carotenoids over the original Golden Rice with a total carotenoid level of up to 37 μg/g dw in the endosperm of which 31 μg/g was β‐carotene. A number of other important food crops have been or are being developed to enhance the level of PVA through genetic modification.

Bananas are the world's most important fruit crop and one of the top 10 crops by production. They are widely grown in the wet tropics and subtropics forming an important dietary component both raw as a dessert fruit and cooked often as the major source of carbohydrate. In a number of countries, bananas are the principal staple food including Uganda where consumption levels average 0.5 kg per person per day rising to around 1 kg per person per day in some regions (Komarek, 2010; Smale and Tushemereirwe, 2007). In East Africa, the staple cultivar is East African highland banana (EAHB) (*M. acuminata* AAA‐EA) prepared primarily by steaming or boiling whereas, in West Africa, plantains are dominant and are usually fried or roasted (Fungo and Pillay, 2011). In both regions, the level of VAD is high. In Uganda, it varies from 15% to 33% in children under 60 months with similar levels in women of childbearing age (UDHS ‐ Uganda Demographic and Health Survey, 2006). Unlike rice endosperm, banana fruit contains PVA and, in some instances, very high levels particularly Fe'i bananas of Micronesia and Papua New Guinea (Englberger *et al*., 2003). Bananas with β‐carotene equivalent (β‐CE) levels of 340 μg/g dw have been reported whereas the dominant dessert banana cv ‘Cavendish’ has between 1 and 4 μg/g dw β‐CE and the EAHB clone, ‘Nakitembe’, has approximately 10 μg/g β‐CE dw (Englberger *et al*., 2006a; Mbabazi, 2015). Unfortunately, domesticated bananas have very low male and female fertility rendering conventional breeding extremely difficult. Thus, the introgression of the high PVA traits of Fe'i bananas for instance into farmer preferred EAHB selections would be practically impossible. However, genetic modification of bananas is well established.

Here, we report the ‘proof‐of‐concept’ technology required towards the generation of PVA‐biofortified EAHB varieties in Uganda. The ‘Cavendish’ dessert banana was genetically modified, and greatly enhanced PVA levels were demonstrated in the fruit of plants grown in the field in Australia.

## Results

### The target

At the outset, it was important to identify a target fruit level of β‐carotene equivalents necessary to help alleviate VAD in Uganda. The target was set at delivering 50% of the estimated average requirement (EAR) of vitamin A in vulnerable populations which for children under 60 months is 120 μg/day and for females ranging from 235 μg/day up to 445 μg/day for lactating mothers. An estimated bioconversion 6:1 ratio of β‐carotene equivalents (β‐CE) to vitamin A from cooked banana pulp was used (Bresnahan *et al*., 2012) with an estimated consumption of cooked bananas of 300 g/day for children and 500 g/day for women. The α‐ and β‐carotene retention after steaming or boiling was also estimated at 70% (Mbabazi, 2015). Using these parameters, banana fruit needed to contain β‐CE levels of at least 20 μg/g dw to achieve 50%of the EAR.

There were three major technical constraints at the commencement of this project that influenced the research strategy: (i) very little information was available regarding the expression of transgenes in bananas generally and more specifically in banana fruit, (ii) the time from transformation to harvestable fruit ranges from 2 to 2½ years, and (iii) it was clearly impractical to take large numbers of transgenic bananas through to fruit in the greenhouse. Therefore, a large number of independent transgenic lines were generated to enable the testing of a wide range of promoter and transgene combinations. Initially, a single plant per transgenic line was planted in the field with, in most instances, between 10 and 30 transgenic lines per construct. For more information, refer to the ‘History of the project’ section of the Supplementary information document.

### Promoter characterization

Three promoters were selected as possible candidates for expressing PVA‐related transgenes in banana fruit, and these were characterized for levels and patterns of expression in transgenic bananas. The promoters included the constitutive maize polyubiquitin promoter (Ubi) and two promoters isolated from banana, the expansin 1 promoter (Exp1) and the ACC oxidase promoter (ACO) which were predicted to be fruit specific. These promoters were fused to the β‐glucuronidase reporter gene (*uidA*), the cassettes transformed into bananas and the transgenic plants established in the field. β‐glucuronidase (GUS) protein levels were measured in the fruit pulp using ELISA; however, this approach could not be used for leaf or peel material because of very high background levels. Therefore, for leaf and peel samples, MUG fluorometric assays were used to estimate enzyme activity rather than protein levels.

**Figure 1:**
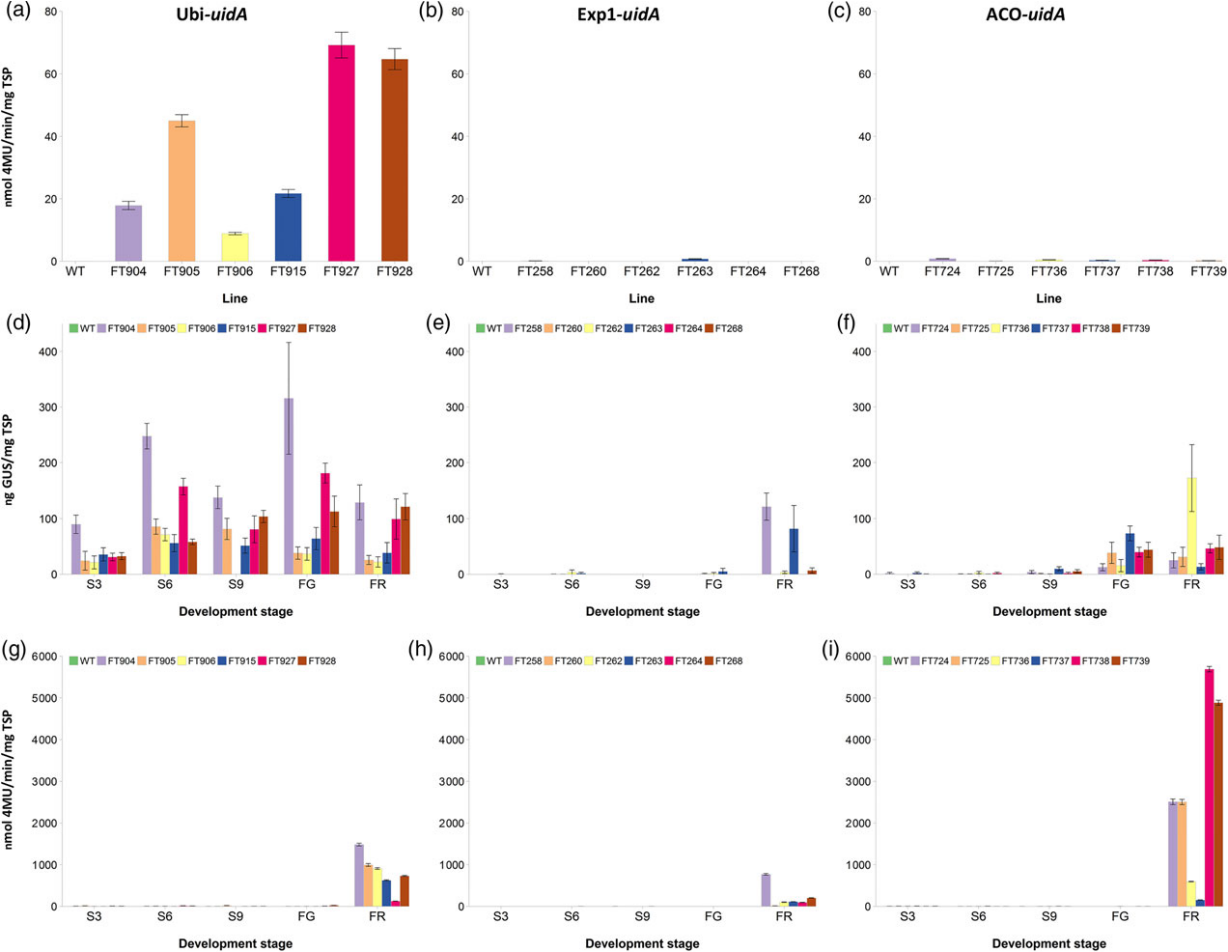
Analysis of promoter activity in wild‐type and transgenic Cavendish banana lines. GUS activity was measured in leaf (a, b, c), and peel (g, h, i), while GUS protein concentration was measured in pulp tissue (d, e, f). Ubi promoter (a, d, g); Exp1 promoter (b, e, h); and ACO promoter (c, f, i). WT, wild‐type FT432. S3, S6 and S9 represent 3, 6 and 9 weeks post‐bunch emergence, respectively. FG, full green and FR, full ripe. Error bars: ±SD.

GUS activity was measured in the leaves of six independent transgenic lines for each promoter. As expected, there were high but variable levels of GUS activity in the leaves of all six plants where *uidA* was under the control of the Ubi promoter (Figure 1a). In the leaves of the wild‐type control plant and plants where *uidA* was under the control of either the Exp1 or ACO promoter, there was undetectable to negligible GUS activity (Figure 1b and c).

Pulp samples from the fruit of the same lines described above were collected at 3, 6, 9 and 12 weeks post‐bunch emergence (S3, S6, S9 and S12) and also at ‘full green’ (FG), when the bunches were harvested, and ‘full ripe’ (FR). The FG stage is equivalent to the stage when cooking bananas are harvested in Uganda. GUS protein was not detected in the wild‐type at any fruit development stage. In contrast, appreciable but varying levels of GUS protein were detected in the pulp of banana fruit from S3 through to FR in the six Ubi‐*uidA* lines (Figure 1d) confirming the constitutive nature of the Ubi promoter. No reproducible trend in GUS protein levels across the six lines was observed as fruit matured from S3 to FR except that average protein accumulation was lowest at the earliest stage, S3. In the six Exp1‐*uidA* lines examined, no appreciable GUS expression was detected in fruit pulp from S3 through to FG with the exception of FR fruit from lines FT258 and FT263 (Figure 1e). This indicated that the Exp1 promoter is activated very late during fruit development. Very low levels of GUS expression were detected in the fruit pulp of the ACO‐*uidA* lines from S3 to S9 (Figure 1f). With the exception of line FT736 which peaked fourfold higher than any other line, the overall trend was that GUS expression slowly increased from S9 through to FG and plateaued at FR. These results indicated that the ACO promoter was activated earlier than Exp1 during fruit development and more consistently at the FG and FR stages (Figure 1e and f).

The pattern of Ubi driven GUS expression in fruit peel was unexpectedly different from fruit pulp with low expression from S3 to FG followed by a substantial increase at FR (Figure 1g). In the peel of Exp1‐*uidA* lines, the pattern of GUS expression was similar to that observed in the fruit pulp (Figure 1h). ACO‐*uidA* lines had very little GUS expression in the peel up to FG; however, there was a dramatic increase to levels higher than either the Ubi or Exp1 lines at FR (Figure 1i).

### PVA analysis: plant and ratoon crops

Domesticated bananas grow as a perennial crop. The initial plant, the plant crop, develops a corm and a pseudostem from which the original bunch is produced. After the bunch is harvested, the pseudostem dies and is replaced by a second pseudostem producing the first ratoon crop which develops from a sucker originating from the corm. Similarly, a second ratoon crop is produced and so on.

The same three promoters used to assess GUS expression were used to drive the expression of three carotenoid biosynthesis transgenes. These transgenes were the phytoene synthase 1 gene from maize (*ZmPsy1*) used in Golden Rice 2, a phytoene synthase 2a gene isolated from the Fe'i banana cultivar Asupina (*MtPsy2a*) (Mlalazi *et al*., 2012) and the bacterial phytoene desaturase gene *PaCrtI* also used in Golden Rice 2. *ZmPsy1* and *MtPsy2a* were transformed into Cavendish banana singly and in combination with *PaCrtI*.

A total of 244 transgenic lines were confirmed to contain the respective PVA transgene(s) by PCR. Southern blot analyses showed that the transgene copy number varied from one to more than 10 copies (Figure S1 and S2). For each line, a single plant was established in the field together with 50 non‐transgenic control plants. This initial randomized trial was designated Field Trial 1 (FT‐1), and the plant crop was assessed over a period of 16 months. Forty‐eight transgenic lines either died or were stunted and did not produce fruit. Therefore, fruit was harvested from a total of 196 transgenic lines of which 153 samples were selected for the initial plant crop fruit analysis (Table 1). Fruit was harvested at FG, ripened to FR and sampled at both stages. The sample used for the initial PVA level screen consisted of a single fruit taken from the middle of the bunch from each of 153 transgenic lines together with the equivalent sample from the fruit of 15 non‐transgenic control plants. Following lyophilization and total carotenoids extraction, each sample was analyzed by HPLC and β‐CE levels calculated. The most important outcomes of this initial screening included: (i) there was obvious variation between individual control plants and also variation within lines with the same promoter‐transgene combination, (ii) the highest expressing transgenic line for each of the different transgenes or combinations of transgenes contained higher levels of β‐CE than the highest control plants except those lines containing Exp1‐*PaCrtI* or Ubi‐*PaCrtI* alone or where Ubi‐*PaCrtI* was combined with Exp1‐*MtPsy2a* or Exp1‐*ZmPsy1*, (iii) in all transgenic lines and controls, the fruit contained higher levels of α‐carotene than β‐carotene (Figure S3), and (iv) *PaCrtI* alone had little effect on fruit PVA levels when driven by either the Exp1 promoter or the Ubi promoter; as such, none of the single transgene *PaCrtI* lines were progressed through for further analysis.

**Table 1:** Data summary from the transgenic banana field trial

**Promoter‐transgene**	**Number of plants in the field**	**Number of plants harvested**	**Number of plants analyzed (first cut)** a	**β‐CE in FG fruit (μg/g dw)**		**β‐CE in FR fruit (μg/g dw)**	
				**Range**	**Average**	**Range**	**Average**
Wild‐type	50	50	15	0.6–3.8	1.5	0.8–5.8	3.1
Exp1‐*MtPsy2a*	33	28	28	1.2–8.6	3.3	0.8–10.0	2.8
Exp1‐*MtPsy2a* + Ubi‐*PaCrtI*	13	8	5	0.2–2.3	1.0	1.3–3.4	2.1
Exp1‐*ZmPsy1*	32	26	26	1.2–4.6	2.6	1.3–15.2	4.5
Exp1‐*ZmPsy1*+ Ubi‐*PaCrtI*	18	11	8	0.2–1.8	1.0	1.5–3.6	2.4
Exp1‐*ZmPsy1*+ Exp1‐*PaCrtI*	7	7	2	–	–	2.1–11.0	6.5
Exp1‐*PaCrtI*	29	26	3	–	–	1.7–2.6	2
ACO‐*MtPsy2a*	30	29	28	11.2–15.3	13.3	3.4–13.4	8.3
ACO‐*ZmPsy1*	30	18	18	5.4–10.9	7.6	2.5–24.6	9.0
ACO‐*ZmPsy1*+ Exp1‐*PaCrtI*	6	4	3	10.6–25.7	17.1	7.8–16.7	13.2
Ubi‐*MtPsy2a*	9	7	7	1.4–19.1	6.8	3.5–16.1	7.4
Ubi‐*ZmPsy1*	10	5	5	0.3–13.6	6.1	1.0–16.1	7.9
Ubi‐*PaCrtI*	27	27	20	–	–	1.0–3.7	1.8
Total	294	246	168	–	–	–	–

a First cut relates to an initial screening of the fruit of transgenic banana lines done by HPLC and using only a single fruit collected from the middle position of the bunch. FG, full green and FR, full ripe.

From this initial plant crop screen, 63 transgenic lines were selected for more comprehensive analysis. The selection included lines representing high, average and low PVA accumulation. As preliminary data revealed considerable variation in PVA accumulation across the bunch (data not shown), carotenoids were extracted from a composite sample including equal amounts of fruit taken from the top, middle and bottom of the bunch. Further, although fruit samples from the selected lines were to be analyzed across three generations (plant crop followed by first and second ratoon crops), the trial was hit by a severe cyclone in February 2011. As a consequence, all lines were blown over resulting in fruit from some lines in either the first or the second ratoon crops not being available for analysis.

For this project, the PVA levels at FG were considered more important as cooking bananas are harvested at this stage in Uganda. However, FR data were also collected as cooking bananas are usually consumed over a number of days post‐harvest. The β‐CE levels in FG and FR fruit from each of the selected 63 transgenic lines are presented in Table 2. Where expression of *MtPsy2a* or *ZmPsy1* was controlled by the Exp1 promoter, the level of β‐CE in the plant crop and first ratoon increased from FG to FR in 43 of 53 samples analyzed (81%) (Table 2). A similar increase was seen in 82% (14 of 17 samples) of the analyzed samples where the Ubi promoter was used to drive the expression of the same transgenes. However, when ACO was used as a promoter, this number reduced to 50% (13 of 26 samples).

**Table 2:** PVA carotenoid concentration in the fruit pulp of selected wild‐type and transgenic Cavendish banana lines across four generations

**Promoter‐transgene**	**Line #**	**Plant crop**		**1st ratoon crop**		**2nd ratoon crop**		**Sucker crop**	
		**FG**	**FR**	**FG**	**FR**	**FG**	**FR**	**FG**	**FR**
		**β‐carotene equivalents (μg/g dw)**							
Wild‐type	Average (n≥ = 6)	2.6	3.1	2.2	2.2	1.7	2.3	6.0	7.4
Exp1‐*MtPsy2a*	FT246	7.3	9.3	8.3	8.5	NA	NA	18.2	19.6
	FT544	3.4	4.6	NA	NA	NA	NA	NA	NA
	FT545	3.4	4.7	NA	NA	NA	NA	NA	NA
	FT342	2.8	4.2	7.4	4.6	NA	NA	10.6	9.0
	FT335	2.5	4.0	2.4	3.3	NA	NA	NA	NA
	FT343	2.3	3.8	2.9	3.4	NA	NA	NA	NA
	FT242	2.2	3.7	2.0	2.0	NA	NA	8.6	8.0
	FT233	2.0	3.3	1.9	2.6	NA	NA	NA	NA
	FT341	1.4	3.1	1.8	2.0	NA	NA	9.3	8.1
Exp1‐*MtPsy2a* + Ubi‐*PaCrtI*	FT244	1.5	2.8	2.6	1.9	NA	NA	NA	NA
	FT245	0.9	1.8	1.8	4.6	NA	NA	NA	NA
	FT220	0.7	1.4	1.7	1.9	NA	NA	NA	NA
	FT232	0.2	1.9	1.8	1.5	NA	NA	NA	NA
Exp1‐*ZmPsy1*	FT534	9.3	11.9	NA	NA	NA	NA	NA	NA
	FT536	9.1	8.9	NA	NA	NA	NA	NA	NA
	FT317	6.6	7.9	NA	NA	NA	NA	11.4	15.6
	FT192	4.3	6.4	NA	NA	NA	NA	20.3	31.2
	FT538	3.8	9.4	NA	NA	NA	NA	10.9	14.8
	FT187	3.5	5.3	1.8	1.8	9.5	9.9	7.0	11.0
	FT311	2.9	6.1	3.1	2.5	NA	NA	NA	NA
	FT201	2.3	3.2	1.9	3.2	NA	NA	9.1	15.0
	FT319	1.9	3.2	3.5	2.6	NA	NA	NA	NA
	FT318	1.5	2.7	1.9	2.4	NA	NA	NA	NA
	FT210	1.3	2.8	1.2	2.5	NA	NA	NA	NA
Exp1‐*ZmPsy1* + Ubi‐*PaCrtI*	FT217	1.8	2.4	1.5	2.4	NA	NA	NA	NA
	FT196	1.6	4.2	2.9	2.5	NA	NA	NA	NA
	FT207	1.5	3.5	2.4	2.7	NA	NA	NA	NA
	FT195	1.0	2.6	NA	NA	NA	NA	NA	NA
	FT208	0.8	2.7	2.1	2.1	NA	NA	NA	NA
	FT205	0.7	2.6	1.0	7.4	NA	NA	NA	NA
	FT213	0.4	1.8	NA	NA	NA	NA	NA	NA
ACO‐*MtPsy2a*	FT504	16.6	12.0	12.1	11.7	NA	NA	20.0	24.7
	FT518	15.9	10.7	NA	NA	NA	NA	23.1	35.9
	FT511	9.4	7.3	5.3	6.0	NA	NA	14.4	13.6
	FT508	9.2	7.1	4.2	3.8	NA	NA	15.6	19.0
	FT498	8.9	9.9	8.6	8.4	NA	NA	NA	NA
	FT506	6.0	5.9	NA	NA	NA	NA	NA	NA
	FT516	4.2	7.4	NA	NA	NA	NA	NA	NA
	FT497	4.1	10.5	NA	NA	NA	NA	13.4	12.9
ACO‐*ZmPsy1*	FT492	9.4	8.5	NA	NA	NA	NA	NA	NA
	FT467	7.3	10.4	NA	NA	NA	NA	10.5	13.4
	FT468	NA	NA	7.3	6.6	NA	NA	NA	NA
	FT475	4.3	10.5	NA	NA	NA	NA	20.7	22.0
	FT493	4.3	18.7	7.0	6.0	NA	NA	NA	NA
	FT476	2.9	5.4	4.5	4.7	NA	NA	NA	NA
	FT487	2.7	15.1	NA	NA	NA	NA	NA	NA
	FT479	2.7	13.6	NA	NA	NA	NA	20.5	16.2
	FT483	1.7	9.1	NA	NA	NA	NA	18.8	16.9
ACO‐*ZmPsy1* + Exp1‐*PaCrtI*	FT584	17.1	11.2	NA	NA	NA	NA	27.0	32.8
	FT587	11.5	NA	NA	NA	NA	NA	21.5	22.2
	FT588	7.7	10.1	NA	NA	NA	NA	NA	NA
	FT585	7.3	5.6	NA	NA	NA	NA	12.2	11.5
Ubi‐*MtPsy2a*	FT328	18.7	18.9	NA	NA	NA	NA	NA	NA
	FT324	11.7	16.1	NA	NA	26.6	33.4	55.0	50.1
	FT294	6.6	9.7	13.5	9.4	13.5	12.0	29.0	25.2
	FT295	5.4	6.1	6.6	6.6	4.8	4.5	10.5	12.0
	FT296	4.2	6.0	5.6	4.1	12.5	13.1	NA	NA
	FT327	3.0	4.9	NA	NA	NA	NA	NA	NA
	FT330	2.5	4.9	3.6	4.0	NA	NA	11.9	15.1
Ubi‐*ZmPsy1*	FT287	13.4	15.8	NA	NA	24.3	21.8	39.7	60.9
	FT309	11.9	14.7	NA	NA	40.4	39.3	46.9	18.5
	FT298	0.7	2.5	1.1	1.3	NA	NA	NA	NA
	FT302	0.6	1.5	1.4	2.0	NA	NA	NA	NA

FG, full green and FR, full ripe.

**Figure 2:**
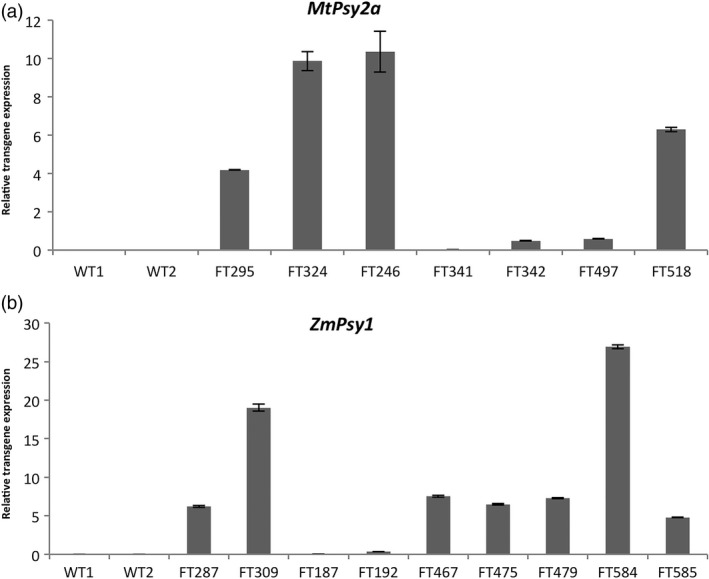
Analysis of mRNA expression levels in the FG fruit pulp of selected transgenic Cavendish banana lines by qRT‐PCR. (a) *MtPsy2a* lines and (b) *ZmPsy1* lines. FG, full green. WT, wild‐type with WT1 = FT167 and WT2 = FT430. Values are normalized expression levels ± SEM.

In the plant crop, there were no lines that met the target level of 20 μg/g dw β‐CE where the composite sample of three fruit per bunch was analyzed. The highest β‐CE level at FG was 18.7 μg/g dw in Ubi‐*MtPsy2a* line FT328 that subsequently died. In addition, only eight lines had PVA levels greater than 10.0 μg/g dw β‐CE at FG. These were two Ubi‐*ZmPsy1* lines (FT287 and FT 309 with 13.4 and 11.9 μg/g dw β‐CE, respectively), two Ubi‐*MtPsy2a* lines (FT328 and FT324 with 18.7 and 11.7 μg/g dw β‐CE, respectively), two ACO‐*MtPsy2a* lines (FT504 and FT518 with 16.6 and 15.9 μg/g dw β‐CE, respectively) and two ACO‐*ZmPsy1 *+* * Exp1‐*PaCrtI* lines (FT584 and FT587 with 17.1 and 11.5 μg/g dw β‐CE, respectively). To investigate whether a correlation existed between transgene expression levels and accumulation of carotenoids, the expression of the *ZmPsy1* and *MtPsy2a* transgenes was determined in the FG fruit of a selection of lines using RT‐PCR (Figure S4 and S5) and qRT‐PCR (Figure 2). *MtPsy2a* line FT246 had the highest relative expression of the transgene followed by line FT324, FT518 and FT295 (Figure 2a). Expression was considerably lower in the other three lines tested. Expression of *ZmPsy1* was highest in line FT584 followed by FT309 while similar, but lower expression was seen in lines FT287, FT467, FT475, FT479 and FT585 (Figure 2b). Lines FT187 and FT192 had low expression.

When FG fruit from the next generation (first ratoon crop) were analyzed, 68% (23 of 34) of samples showed an increase in β‐CE from the plant crop (Table 2). Interestingly, every line analyzed that contained either Ubi‐*ZmPsy1* or Ubi‐*MtPsy2a* showed an increased accumulation in β‐CE from the plant crop. However, again, no line accumulated over the 20 μg/g dw β‐CE target level. FG fruit from two lines had PVA levels above 10 μg/g dw β‐CE in the ratoon crop: ACO‐*MtPsy2a* line FT504 with 12.1 μg/g dw β‐CE down from 16.6 μg/g dw β‐CE in the plant crop and Ubi‐*MtPsy2a* line FT294 with 13.5 μg/g dw β‐CE up from 6.6 μg/g dw β‐CE in the plant crop. Due to the impact of the 2011 cyclone, only seven lines could be assessed in the second ratoon. FG fruit from three of these lines accumulated above target levels of PVA. Ubi‐*ZmPsy1* line FT309 had FG fruit with 40.4 μg/g dw β‐CE, more than double the target, while Ubi‐*MtPsy2a* line FT324 and Ubi‐*ZmPsy1* FT287 accumulated 26.6 and 24.3 μg/g dw β‐CE, respectively (Table 2).

**Figure 3:**
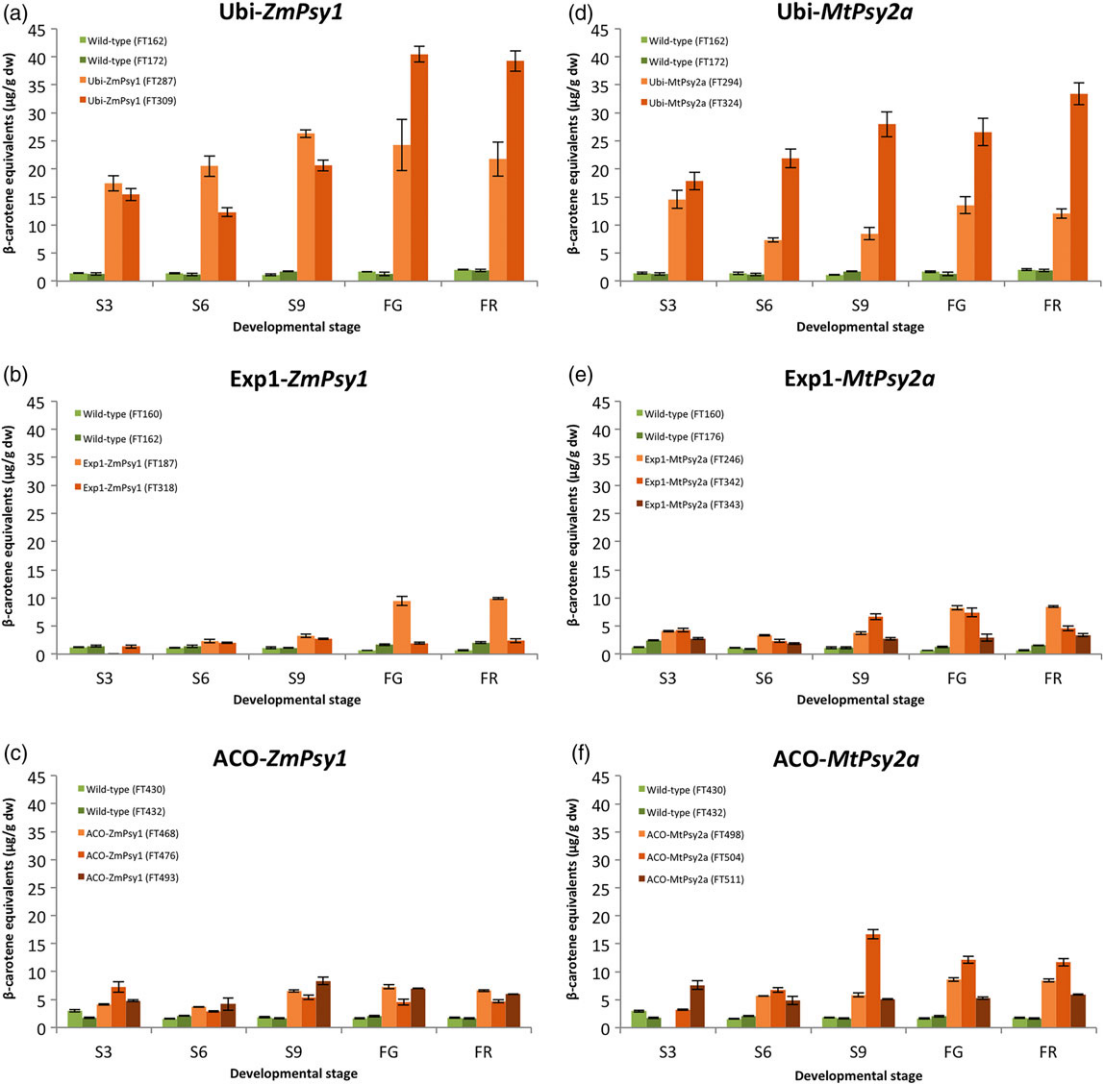
PVA carotenoid accumulation in the pulp of wild‐type and selected transgenic Cavendish banana lines during fruit development. S3, S6 and S9 represent 3, 6 and 9 weeks post‐bunch emergence, respectively. FG, full green and FR, full ripe. Error bars = ±SD.

Carotenoid accumulation throughout fruit development was also monitored in selected lines from each of the single promoter‐*Psy* combinations in the second ratoon crop at 3, 6 and 9 weeks post‐bunch emergence as well as FG and FR (Figure 3). For the four lines where the transgene was under the control of the Ubi promoter, PVA levels were elevated (above 15 μg/g dw β‐CE) from the earliest fruit collection time point (S3) irrespective of whether the transgene was *ZmPsy1* or *MtPsy2a* (Figure 3a and d). For the two lines with the highest PVA levels, the general trend was increasing PVA during fruit development to a maximum at FG for Ubi‐*ZmPsy1* line FT309 or at FR for Ubi‐*MtPsy2a* line FT324. In contrast, accumulated PVA levels in the fruit of Exp1‐*ZmPsy1* and Exp1‐*MtPsy2a* lines remained below 5 μg/g dw β‐CE from the emergence of the bunch all the way through to S9 (with the exception of line FT342 which accumulated 6.6 μg/g dw β‐CE at S9) followed by an increase towards maturity with a maximum of 9.9 μg/g dw β‐CE at FR in Exp1‐*ZmPsy1* line FT187 (Figure 3b and e). During fruit development of lines containing ACO‐*ZmPsy1*, PVA levels were lowest at S3 and S6 for two of the lines but moderately higher than the Exp1‐*ZmPsy1* lines at those stages (Figure 3c). PVA levels peaked for those same two lines at either S9 or FG. In contrast, the highest PVA level in line FT476 was at S3. For the ACO‐*MtPsy2a* lines, again one line, FT511, had maximum PVA accumulation at S3, while the other two lines had maximums at S9 or FG (Figure 3f). Overall, the PVA accumulation pattern during fruit development reflected the expression profiles previously observed in transgenic lines where the same three promoters were used to drive the expression of *uidA* (Figure 1). The constitutive Ubi promoter provided consistent stronger expression throughout fruit development followed by the ACO promoter and finally Exp1.

**Figure 4:**
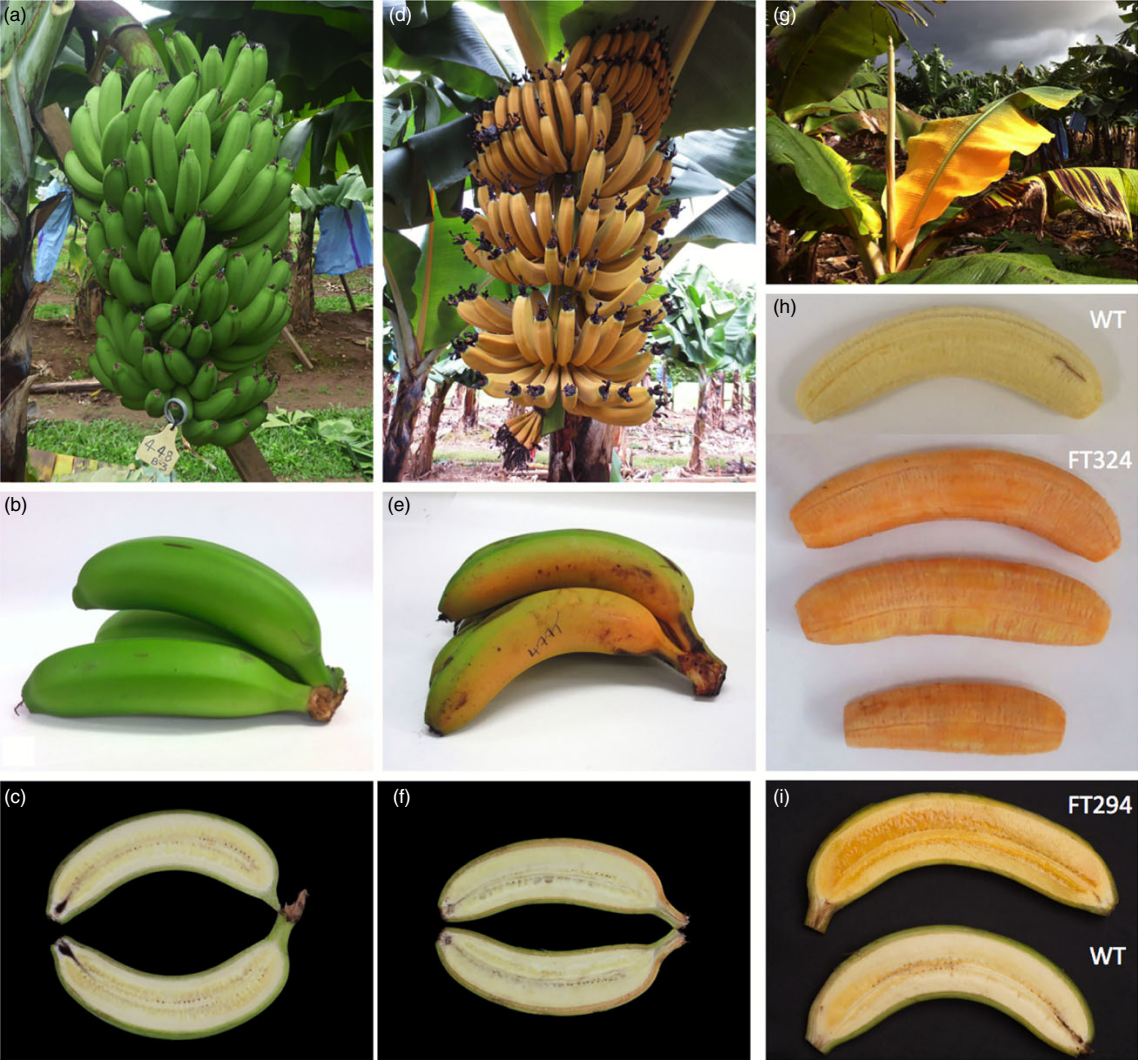
Characteristic phenotypes observed in wild‐type and transgenic Cavendish banana lines. (a) Bunch from wild‐type line FT448; (b) fruit from wild‐type line FT448; (c) longitudinal section of fruit from wild‐type line FT448; (d) immature bunch from ACO‐*ZmPsy1* line FT477; (e) fruit from ACO‐*ZmPsy1* line FT477; (f) longitudinal section of fruit from ACO‐*ZmPsy1* line FT477; (g) Exp1‐*ZmPsy1* line FT192; (h) Ubi‐*MtApsy2a* line FT324; and (i) Ubi‐*MtApsy2a* line FT294. WT, wild‐type.

During the plant and ratoon crops, the phenotype of each plant was recorded at regular intervals from planting to bunch harvest. None of the 50 wild‐type control plants showed altered phenotypes and fruit developed normally (Figure 4a–c). The presence of the *PaCrtI* transgene did not appear to affect phenotype. However, three categories of altered phenotypes were observed in the transgenic lines: stunting, ‘golden leaf’ and ‘golden bunch’. For the ‘golden leaf’ phenotype, the youngest leaf would consistently unfurl with a bright yellow colour (‘golden’) and progressively turn to green as it matured (Figure 4g). Fruit on the ‘golden bunch’ emerged bright orange instead of green (Figure 4d). As the fruit matured and filled, it progressively turned greener to a mixture of green and orange at harvest (Figure 4e and f). Fruits with increase PVA levels displayed a pulp ranging from deep yellow to bright orange (Figure 4h and i). Of the original 244 transgenic lines planted in the trial, 65 had the ‘golden leaf’ phenotype of which 29 were also stunted; 29 had the ‘golden bunch’ phenotype. The ‘golden leaf’ and ‘golden bunch’ phenotypes were highly transgene dependent where lines with those phenotypes invariably contained the *ZmPsy1* transgene in contrast to lines containing the *MtPsy2a* transgene. The ‘golden leaf’ phenotype was observed in 27 of 57 (50%) lines observed carrying Exp1‐*ZmPsy1* alone or together with either Exp1‐*PaCrtI* or Ubi‐*PaCrtI*. More importantly, the ‘golden bunch’ phenotype which only occurred in ACO‐*ZmPsy1* or ACO‐*ZmPsy1 *+* * Exp1‐*PaCrtI* lines was recorded in 94% (29 of the 31) of the lines assessed. In contrast, across all 85 lines containing *MtPsy2a*, only 7 (8%) had the ‘golden leaf’ phenotype and none displayed a ‘golden bunch’.

The β‐CE levels in the pulp and peel of FG and FR fruit from lines with the ‘golden bunch’ phenotype were analyzed and compared with the levels in fruit from phenotypically normal lines. In the FG fruit from four lines of ACO‐*MtPsy2a*, the levels of β‐CE in the peel were similar to those in the three non‐transgenic controls (Table 3). However, the peel β‐CE levels in four ACO‐*ZmPsy1* containing lines were more than ninefold higher than in fruit peel from the control lines (Table 3). Importantly, the peel β‐CE levels in the ACO‐*ZmPsy1* lines did not influence fruit pulp β‐CE levels as the four ACO‐*ZmPsy1* lines had a similar range of β‐CE levels in their fruit pulp at FG to fruit from the four ACO‐*MtPsy2a* lines (Table 3).

**Table 3:** PVA carotenoid concentration in the fruit pulp and peel of wild‐type and selected transgenic Cavendish banana lines

**Promoter‐transgene**	**Line**	**β‐CE in pulp (μg/g dw)**		**β‐CE in peel (μg/g dw)**	
		**FG**	**FR**	**FG**	**FR**
Wild‐type	FT166	6.6	7.3	74.2	32.1
Wild‐type	FT430	6.5	8.3	126.5	45.4
Wild‐type	FT448	8.0	9.4	99.5	38.8
ACO‐*MtPsy2a*	FT504	20.0	24.7	108.3	72.7
ACO‐*MtPsy2a*	FT508	15.6	19.0	68.3	72.6
ACO‐*MtPsy2a*	FT511	14.4	13.6	105.7	75.0
ACO‐*MtPsy2a*	FT518	23.1	35.9	129.2	131.2
ACO‐*ZmPsy1*	FT467	10.5	13.4	742.1	703.2
ACO‐*ZmPsy1*	FT475	20.7	22.0	1112.2	1132.6
ACO‐*ZmPsy1*	FT479	20.5	16.2	740.9	833.0
ACO‐*ZmPsy1*	FT483	18.8	16.9	1155.1	837.3

FG, full green and FR, full ripe. All samples were collected from the sucker crop.

### PVA analysis: sucker crop

Following the initial plant and ratoon crop assessment with single plants per line, 30 lines selected from seven promoter/transgene combinations and five wild‐type lines were multiplied through suckering to a maximum of 10 replicates per line. A total of 239 transgenic and 48 wild‐type plants derived from suckers were planted in a second field trial (FT‐2). Each plant was harvested, and PVA levels were measured in the fruit at FG and FR by HPLC.

**Figure 5:**
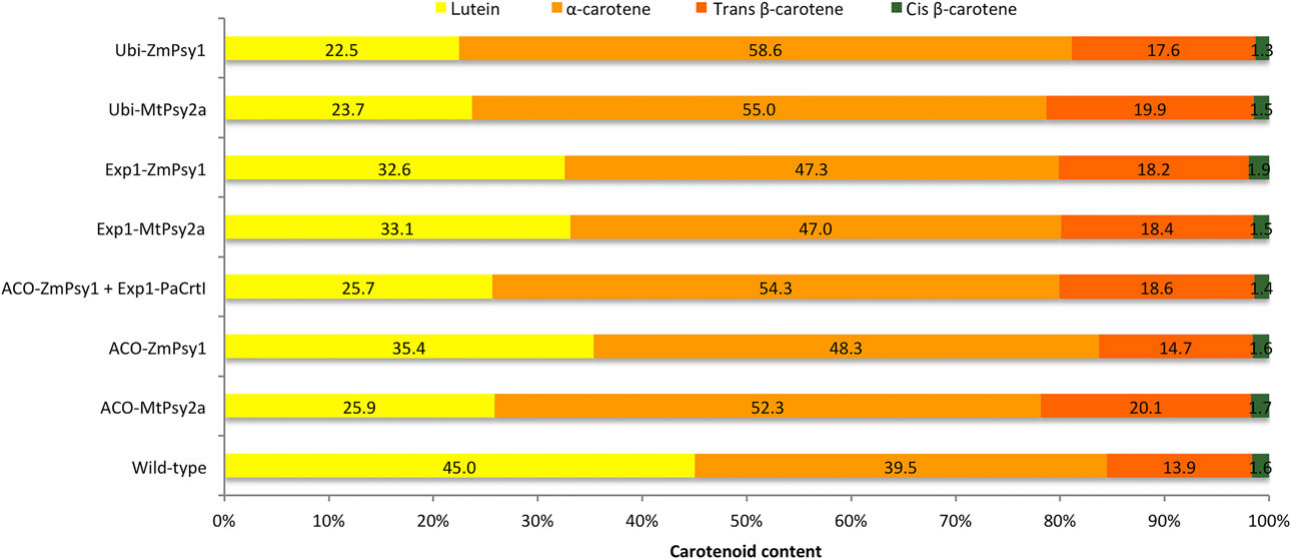
Percentage accumulation of individual carotenoids in the fruit pulp of wild‐type and transgenic bananas. Percentage (%) carotenoid content calculated based on total carotenoid content measured in the pulp of full green fruit collected from the sucker crop. Biological replicates: wild‐type (n = 5), ACO‐*MtPsy2a* (n = 5), ACO‐*ZmPsy1* (n = 4), ACO‐*ZmPsy1 *+* *Exp1‐*PaCrtI* (n = 3), Exp1‐*MtPsy2a* (n = 4), Exp1‐*ZmPsy1* (n = 5), Ubi‐*MtPsy2a* (n = 4) and Ubi‐*ZmPsy1* (n = 2). All samples were analyzed in three technical replicates.

In all transgenic and wild‐type lines, the averaged fruit PVA level was higher in the sucker crop than in either the plant or ratoon crops. The highest average PVA level was 55.0 μg/g dw β‐CE found in the FG fruit of Ubi‐*MtPsy2a* line FT324 (Table 2) with one individual plant of this line reaching 73.8 μg/g dw β‐CE (data not shown). Fruit from this plant had bright orange pulp compared with the fruit of non‐transgenic control plants (Figure 4h). Of the 27 transgenic lines shown in Table 2, 11 had fruit PVA levels equal to or greater than the 20 μg/g dw β‐CE target. However, the fruit PVA levels in the wild‐type controls were also higher than in the plant and ratoon crops with an average of 6.0 μg/g dw β‐CE. Interestingly, the top four sucker crop lines all contained an Ubi‐*Psy* promoter/transgene combination. Furthermore, six of 12 ACO promoter lines had PVA levels equal to or greater than target level, compared to only one Exp1 promoter line of 9 (Table 2). Analysis of the carotenoid composition of fruit pulp from wild‐type as well as transgenic fruit in the sucker crop revealed that, like the plant crop fruit, samples contained higher levels of α‐carotene than β‐carotene and in similar proportions (Figure 5).

### Variation in fruit PVA levels in wild‐type banana

**Figure 6:**
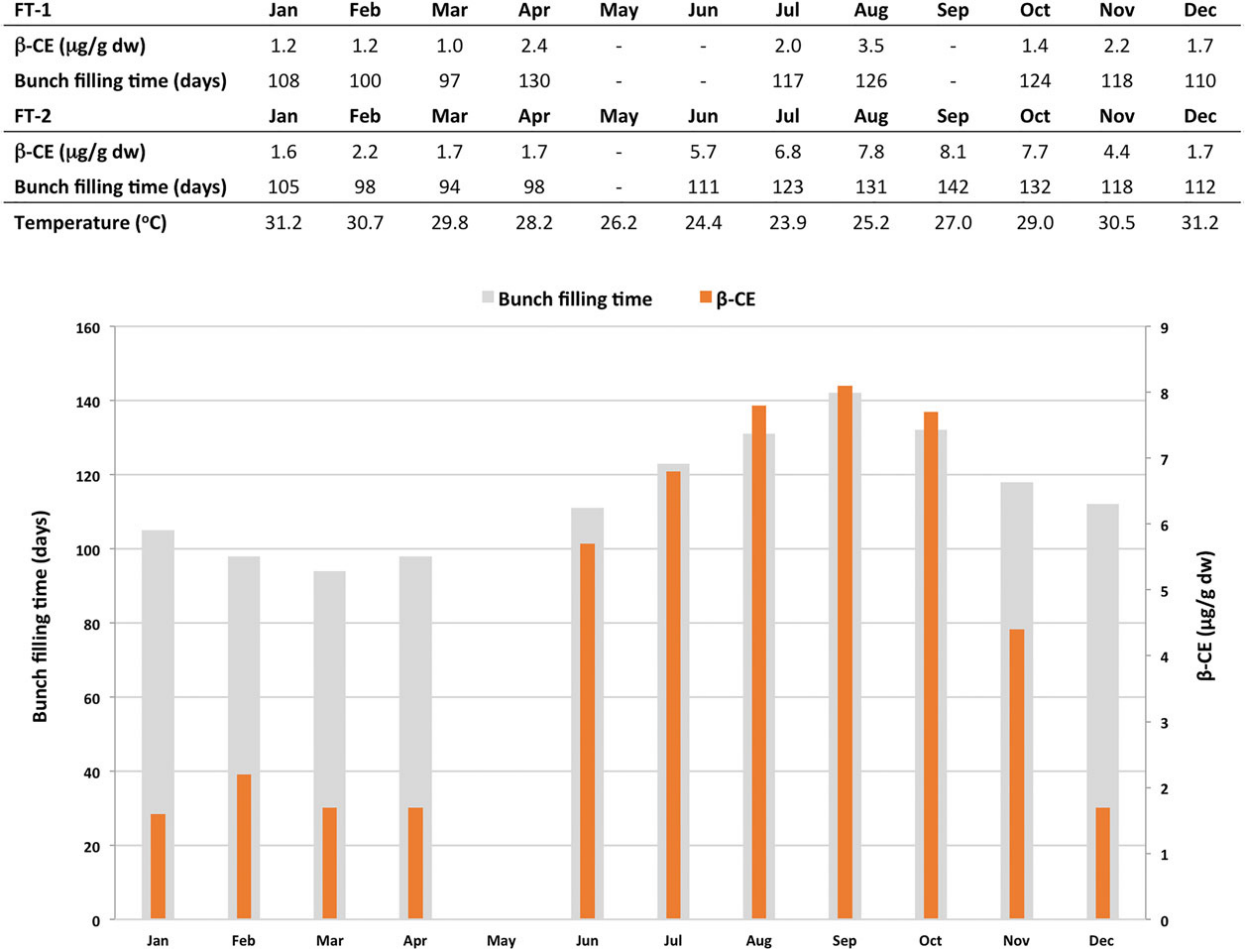
Influence of temperature and time to fruit maturity on the concentration of PVA carotenoids in the FG pulp of wild‐type Cavendish banana. For each month, β‐CE levels and time from bunch emergence to harvest were averaged from all samples collected. FG, full green.

The average PVA levels in the FG fruit of non‐transgenic control plants varied considerably throughout the two field trials from a low of 1.0 μg/g dw β‐CE in March‐harvested fruit in the plant crop of FT‐1 to 8.1 μg/g dw β‐CE in September‐harvested fruit of FT‐2 (Figure 6). When analyzed together, a strong correlation was observed between the level of accumulated PVA in the fruit and the number of days from bunch emergence to harvest (bunch filling time). Indeed, fruit harvested in March had the shortest bunch filling time (94–97 days) and the lowest accumulated PVA level in the fruit compared with 142 days for September‐harvested fruit which had the highest fruit PVA levels (Figure 6). Longer bunch filling time and associated increased levels of PVA also appeared temperature dependent where fruit maturing during the cooler months had higher levels of accumulated PVA.

## Discussion

Micronutrient deficiencies remain a substantial burden on the public health of populations particularly in developing countries. The ‘poorest of the poor’ are particularly impacted by micronutrient deficiencies because of their increasing dependence on nutrient poor single staple crops for the majority of their calorific intake (Muthayya *et al*., 2013). There have been significant inroads into reducing VAD in children aged 6 to 59 months where worldwide prevalence has fallen from 39% in 1991 to 29% in 2013 in low‐ and middle‐income countries (Stevens *et al*., 2015). However, VAD prevalence in children age 6–59 months in Uganda has increased from 20% in 2006 to 38% in 2011 and a similar increase was seen from 19% to 36% in women age 15–49 years (UDHS ‐ Uganda Demographic and Health Survey, 2006, 2011). In Uganda, the staple crop is cooking bananas, more specifically East African highland bananas (EAHB). These bananas are a group of very similar triploid clones of *Musa acuminata* with low male and female fertility levels.

Therefore, a metabolic engineering strategy that could provide the basis for elevating fruit PVA levels in EAHB was developed. Previously, the most common strategy used to achieve transgenic elevated PVA has been the Golden Rice 2 strategy where seed expression of both *ZmPsy1* and *PaCrtI* has been reported to significantly increase seed PVA in rice (Paine *et al*., 2005), maize (Naqvi *et al*., 2009; Zhu *et al*., 2008), wheat (Cong *et al*., 2009) and sorghum (Lipkie *et al*., 2013) with levels up to 31 μg/g dw in rice and 59.3 μg/g dw in maize. A range of other transgenes have also been tested either alone or in combinations including *PaCrtB* in potato (Ducreux *et al*., 2005), tomato (Fraser *et al*., 2002), canola (Shewmaker *et al*., 1999), maize (Aluru *et al*., 2008), soya bean (Schmidt *et al*., 2015) and cassava (Sayre *et al*., 2011), *AtDXS* in cassava and sorghum (Lipkie *et al*., 2013; Sayre *et al*., 2011) and the brassica Or gene, *BaOr,* in potato (Li *et al*., 2012).

In the present study, the dominant dessert banana, Cavendish, was used as a model for EAHB as it is also a triploid *M. acuminata*. Two phytoene synthase transgenes were tested with and without co‐expression of *PaCrtI*. Interestingly, over‐expression of phytoene synthase as a strategy to increase PVA had previously only been reported in cereal crops for which the transgene used was always *ZmPsy1* except in Golden Rice 1. In this work, *ZmPsy1* was tested as well as *MtPsy2a*, a banana phytoene synthase gene previously cloned from the fruit of a naturally high PVA Fe'i banana called Asupina (Mlalazi *et al*., 2012). This approach allowed the generation of eleven transgenic lines which produced fruit that contained greater than the target level of 20 μg/g dw β‐CE. The highest level of PVA in the fruit of a single banana plant was from line FT324 (73.8 μg/g dw β‐CE) which averaged at 55.0 μg/g dw β‐CE. This line contained the banana phytoene synthase gene (*MtPsy2a)* under the control of the constitutive ubiquitin promoter. The highest PVA levels with the phytoene synthase transgene under the control of an apparently fruit‐specific promoter with or without co‐expression of *PaCrtI* were also above target and included one ACO‐*ZmPsy1*+ Exp1‐*PaCrtI* line with 27.0 μg/g dw β‐CE, one ACO‐*ZmPsy1* line with 20.7 μg/g dw β‐CE and one ACO‐*MtPsy2a* line with 23.1 μg/g dw β‐CE. The levels of PVA in Line 324 are equivalent to the highest levels obtained in any other crop where a plant phytoene synthase gene has been over‐expressed. In maize transformed with the seed‐expressed *ZmPsy1* and *PaCrtI* transgenes, Zhu *et al*. (2008) and Naqvi *et al*. (2009) both reported levels of 57.4 and 59.3 μg/g dw β‐CE, respectively. However, Schmidt *et al*. (2015) recently reported that soya beans transformed with *PaCrtB* alone under the control of the seed‐specific Le1 promoter accumulated up to 845 μg/g dw β‐carotene in the seed.

Although two banana lines containing ACO‐*ZmPsy1* had above target levels of PVA, nearly all lines transformed with this construct had a ‘golden bunch’ phenotype which was never observed in ACO‐*MtPsy2a* transformed plants. That ACO‐*ZmPsy1* expression should result in a ‘golden bunch’ phenotype while Exp1‐*ZmPsy1* expression resulted invariably in a ‘golden leaf’ phenotype suggests that these two promoters express in tissue other than fruit pulp and most likely in either leaf primordia for Exp1 or ‘bunch’ primordia for ACO. The molecular basis for both the phenotypes has not yet been determined and is under investigation.

An important outcome from this study was that the levels of PVA in most banana lines increased through the ‘generations’ from the plant crop through the first ratoon, second ratoon to finally the sucker crop. Line FT324 had only 11.7 μg/g dw β‐CE in the FG plant crop to 55.0 μg/g dw β‐CE in the sucker crop. Bananas are vegetatively propagated and do not go through a seed phase, and thus, all generations are T_0_. The explanation for the phenomenon of increasing PVA levels with successive vegetative generations is not entirely understood but it did demonstrate that, rather than there being a reduction of expression with successive vegetative generations as a result of transgene silencing, the trait was stable. Further, it is possible that this phenomenon might also occur in other vegetatively propagated crops demonstrating the importance of monitoring transgenic traits in vegetative propagated crops in the field through multiple ‘generations’.

Two outcomes from this study indicated that the final level of PVA was a result of accumulation through the development of the banana fruit. Firstly, the levels of PVA in non‐transgenic Cavendish fruit were found to vary considerably. Our observations indicated that these variations correlate with time to fruit maturity. Indeed, the highest PVA levels were obtained in fruit with the longest maturity time. This probably accounts for the varying reported levels of fruit PVA levels in cultivars such as Cavendish (Davey *et al*., 2007; Englberger *et al*., 2006b; Fungo and Pillay, 2011) as well as much of the variation observed within transgenic lines in different seasons. This variable was controlled for in the ‘sucker generation’ where all suckers were planted on the same day at the same location. Secondly, the two lines that accumulated the highest PVA concentration contained the phytoene synthase genes under the control of the Ubi promoter. This promoter was previously demonstrated to be active from the earliest stages of banana fruit development in contrast with the late fruit expression from the ACO and Exp1 promoters.

Two constructs containing Ubi‐*MtPsy2a* and ACO‐*MtPsy2a* have been transferred to the National Agricultural Research Organization (NARO) in Uganda and have been transformed into two Ugandan cultivars including EAHB.

## Experimental procedures

### Vector construction

The coding regions of *Musa troglodytarum* x *acuminata* cultivar Asupina phytoene synthase 2a (*MtPsy2a*; GenBank #: JX195659), *Zea mays* cultivar B73 phytoene synthase 1 (*ZmPsy1*; GenBank #: U32636) and *Pantoea ananatis* phytoene desaturase (*PaCrtI*; Genbank #: D90087) genes were used to facilitate PVA enhancement in banana. The *uidA* gene from *Escherichia coli*, encoding the enzyme β‐glucuronidase (GUS), containing a catalase intron (Khanna *et al*., 2004), was used to assess promoter activity. Each of these genes was fused to the *Agrobacterium tumefaciens* nopaline synthase (Nos) 3′ transcription termination regulatory sequence (Depicker *et al.,* 1982). The banana expansin (Exp1; GenBank #: JN172931) and 1‐aminocyclopropane‐1‐carboxylate oxidase (ACO; GenBank #: AF221107) promoters, and the maize polyubiquitin1 promoter (Ubi, Dugdale *et al*., 2000) where characterized in banana using each of them to drive *uidA* expression, and the resulting expression cassettes: Exp1‐*uidA*‐nos, ACO‐*uidA*‐nos and Ubi‐*uidA*‐nos were assembled in the pBIN‐19 (GenBank #: U09365) binary vector backbone. These three promoters were also used to regulate the expression of the *MtPsy2a* and *ZmPsy1* genes, resulting in the production of six expression cassettes in the pCAMBIA‐2300 (GenBank #: AF234315) binary vector backbone. Expression of the *PaCrtI* gene was driven by the Ubi or Exp1 promoter, and the resulting Ubi‐*PaCrtI*‐nos and Exp1‐*PaCrtI*‐nos expression cassettes were assembled in pBIN‐19. Selection of transgenic plants was mediated by the neomycin phosphotransferase II gene (*nptII*; Beck *et al*., 1982) in both pCAMBIA‐2300 and pBIN‐19.

### Plant transformation and regeneration

Transgenic *Musa acuminata* (AAA Group) ‘Dwarf Cavendish’ lines were generated via *Agrobacterium*‐mediated transformation of embryogenic cell suspensions (ECS) using *A. tumefaciens* strain AGL1 (Khanna *et al*., 2004). Binary vectors containing *MtPsy2a* or *ZmPsy1* were used for transformation of banana ECS either alone or in combination with one of the vectors containing *PaCrtI*, while vectors containing *uidA* were all used individually for transformation.

### Plant material and field trials

Transgenic and wild‐type banana lines established in tissue culture were transported to the field trial site at the Department of Agriculture and Fisheries (DAF) South Johnstone Research Facility (Queensland, Australia) according to conditions on the Office of the Gene Technology Regulator (OGTR) licence number DIR109. Plants were acclimatized in soil and grown in a glasshouse for 12 weeks before planting and maintenance in the field according to standard agronomic procedures. The initial field trial (FT‐1) was conducted between 2009 and 2012. Promising transgenic lines and selected wild‐type control lines (up to 10 sucker plants per line) were established for 12 weeks in the glasshouse before being transferred to a second field trial (FT‐2) at the same facility, which commenced in September 2012.

### Field sample collection and processing

Mature green (full green—FG) fruit was harvested from each plant and sent to the Centre for Tropical Crops and Biocommodities (CTCB) laboratory in Brisbane, Australia, within 48 h of harvest. When analysis of developing fruit was required, fruit was taken at 3, 6 and 9 weeks post‐bunch emergence (S3, S6 and S9) prior to harvesting the entire bunch at FG. Fruit was handled under low‐light conditions and was processed either immediately (for FG analysis) or at 7 days post‐exposure (24 h) to ethylene for full ripe (FR) analysis. Representative fruit from the top, middle and bottom of the bunch was received at all stages of fruit development from plants transformed with *Psy* or *CrtI* genes, while fruit from the top of the bunch only was obtained from lines transformed with the *uidA* gene. Leaf samples from the field were collected from the first fully expanded leaf prior to bunch emergence. Prior to further analysis, all samples were freeze‐dried in a Benchtop 4K Freeze Dryer (VirTis^®^) and homogenized in a Mini‐Beadbeater‐8^™^ (Biospec Products) tissue disruptor.

### Nucleic acid isolation

Genomic DNA (gDNA) for PCR and Southern blot analysis was isolated from 50 mg of homogenized freeze‐dried leaf tissue using a modified CTAB method (Stewart and Via, 1993). Isolation of plasmid DNA (pDNA) was done using the Wizard^®^ Plus SV Minipreps DNA Purification System (Promega) according to the manufacturer's instructions. Total RNA was extracted from 50 mg of homogenized freeze‐dried banana fruit tissue essentially as described by Valderrama‐Cháirez *et al*. (2002).

### DNase treatment and complementary DNA (cDNA) synthesis

For cDNA synthesis, 3 μg of total RNA was DNase treated using an RQ1 RNase‐free DNase Kit (Promega). DNA‐free RNA samples (1.8 μg) were reverse‐transcribed to cDNA using an oligo(dT20) primer and the GoScript^™^ Reverse Transcription System (Promega) in 25 μL reactions according to the manufacturer's instructions.

### Polymerase chain reaction (PCR) and reverse‐transcription PCR (RT‐PCR)

Putatively transgenic tissue culture plants were tested under standard PCR conditions using oligonucleotide primers designed to amplify gene fragments spanning the *MtPsy2a*,* ZmPsy1* and *PaCrtI* genes and their respective promoter region and using GoTaq^®^ Green master mix (Promega). Transgene‐positive plants were further PCR tested for the presence of contaminating *Agrobacterium* using *VirC* gene primers (Haas *et al*., 1995). Complementary DNA (cDNA) from PCR‐positive plants was then used in RT‐PCR to verify gene expression.

### Quantitative real‐time PCR (qRT‐PCR)

Quantitative RT‐PCRs were done in a CFX384 Touch^™^ Real‐Time PCR Detection System (Bio‐Rad) using GoTaq qPCR Master Mix (Promega). Each sample was analyzed in three technical replicates in addition to the inclusion of ‘no template’ and ‘RT‐negative’ controls. The following amplification parameters were used: Hot‐Start polymerase activation at 95 °C for 2 min, followed by 45 cycles of 10 s denaturation at 95 °C and 30 s annealing/extension at 60 °C. At the end of the reaction, a dissociation curve was produced from 65 to 95 °C to confirm the specificity of the amplicon from each primer set. Fluorescence was recorded in real time and detected at 470 nm. Relative expression levels were calculated using the CFX Manager 3.1 (Bio‐Rad) software and the ΔCT method (Schmittgen and Livak, 2008). Ct data obtained from target gene of interest (GOI) were normalized using Ct values from the two stable reference genes cyclophilin (*CYP*) and ribosomal protein S2 (*RPS2*). All primers were designed using the Primer3Plus freeware (http://www.bioinformatics.nl/cgi-bin/primer3plus/primer3plus.cgi) (Table S1).

### Southern hybridization

For determination of transgene copy number integration by Southern analysis (Southern, 1975), genomic DNA (10 μg) as well as positive control plasmid DNA (20 ng) were digested for 16 h and 1 h, respectively, by incubation at 37 °C with 20 U of restriction enzyme. Restriction enzymes were selected to only cut once within the binary vector T‐DNA region without cutting the region to which the probe would hybridize. PCR based DIG‐labelled probes (Roche) were designed targeting the coding regions of *MtPsy2a* and *ZmPsy1*. Digested DNA was electrophoresed, blotted and detected under standard Southern blotting conditions (Sambrook and Russell, 2001).

### Carotenoid content quantification

Carotenoids were extracted from banana pulp (200 mg) or peel (25 mg) tissue and analyzed by HPLC as previously described (Buah *et al*., 2016). Total carotenoids and β‐carotene equivalents (β‐CE) were expressed in μg/g dry weight (dw).

### Assessment of promoter activity

β‐glucuronidase (GUS) content was measured in banana pulp tissue (40 mg) by ELISA as per Dugdale *et al*. (2013), while fluorometric quantification of GUS activity was determined in leaf and peel tissue as described by Jefferson *et al*. (1987).

## Conflict of interest

Authors declare no conflict of interest.

## Supporting information


**Figure S1.** Determination of transgene copy number in transgenic Cavendish banana lines by Southern blot analysis.
**Figure S2.** Determination of transgene copy number in transgenic Cavendish banana lines by Southern blot analysis.
**Figure S3.** Representative HPLC chromatogram of the main carotenoids in wild‐type and transgenic Cavendish banana.
**Figure S4.** Transgene expression analysis in selected *MtPsy2a* transgenic Cavendish banana lines by reverse transcriptase‐PCR (RT‐PCR).
**Figure S5.** Transgene expression analysis in selected *ZmPsy1* transgenic Cavendish banana lines by reverse transcriptase‐PCR (RT‐PCR).
**Table S1.** List of oligonucleotide primer sequences used for RT‐PCR and qRT‐PCR.
