# Supplementary material for: Golden bananas in the field: elevated fruit pro‐vitamin A from the expression of a single banana transgene
Source: Plant Biotechnol J. 2016 Dec 20;15(4):520–32. doi: 10.1111/pbi.12650 (PMC5362681; doi:10.1111/pbi.12650)
Supplement: Supplementary file 1 — Figure S1. Determination of transgene copy number in transgenic Cavendish banana lines by Southern blot analysis. Figure S2. Determination of transgene copy number in transgenic Cavendish banana lines by Southern blot analysis. Figure S3. Representative HPLC chromatogram of the main carotenoids in wild‐type and transgenic Cavendish banana. Figure S4. Transgene expression analysis in selected MtPsy2a transgenic Cavendish banana lines by reverse transcriptase‐PCR (RT‐PCR). Figure S5. Transgene expression analysis in selected ZmPsy1 transgenic Cavendish banana lines by reverse transcriptase‐PCR (RT‐PCR). Table S1. List of oligonucleotide primer sequences used for RT‐PCR and qRT‐PCR. [file PBI-15-520-s001.docx]

**Supplementary information**

**Golden bananas in the field: elevated fruit pro-vitamin A from the expression of a single banana transgene**

**History of the project**

The ultimate aim of this project is to develop bananas with elevated levels of pro-vitamin A as a strategy to overcome the unacceptably high levels of vitamin A deficiency in Uganda and other countries where bananas are a staple food. Bananas are essentially perennial. When a banana is planted, a pseudostem comprised of the petioles of leaves grows from the basal corm with the vegetative meristem remaining at the base of the pseudostem. When flowering is triggered, the meristem is pushed up through the centre of the pseudostem, the bunch emerges and progresses through to maturity and is finally harvested. This pseudostem dies back and another new pseudostem, known as the first ratoon, grows from another meristem on the corm. This process can be repeated indefinitely.

The overarching strategy for the project was to develop the technology in Australia and transfer the technology to Uganda where the local bananas would be transformed, trialled, selected and released. This paper describes the development of that technology. In Uganda, the major cultivar is East African Highland banana (EAHB) which is harvested green and cooked by boiling or steaming. EABH are triploid Musa acuminata. In Australia, we used Cavendish bananas as the model for EAHB. Cavendish is traditionally harvested green and ripened before consumption as a raw fruit. It is not normally used as a cooking banana but is also a triploid Musa acuminata.

A target of 50% of the Estimated Average Requirement (EAR) for vitamin A was proposed by the Global Health program within the Bill & Melinda Gates Foundation. Although it is technically achievable, 100% of the EAR is probably not desirable and may discourage the development of a diversified diet. Single staple biofortification is not “the” solution but rather part of the solution to help alleviate micronutrient deficiency of the world’s poor.

There were a number of factors that influenced our approach to developing the technology. Firstly, banana plants are large and it was clearly impossible to take a large number of transgenic events through to fruit harvest in the glasshouse. We therefore applied for and were granted approval from the Australian regulator to take nearly 2000 independent transgenic events to the field with minimal prior characterization. Secondly, it takes at least 3.5 years from transformation to harvesting the first GM fruit in the field. Considering this timeframe, we decided that attempting to identify the best combination(s) of promoter and transgene would be best achieved by testing as many promoter/transgene combinations in parallel. Finally, it certainly wasn’t obvious the expression of which transgenes and promoters would result in elevated fruit pro-vitamin A to the level of the target. Our approach therefore was to test multiple transgenes and multiple promoters and different combinations thereof in parallel. We also decided to test up to 30 independent transgenic lines per construct with one plant per line rather than multiple copies of a transgenic line with fewer lines as we were constrained by the size of the trial. This approach was successful in identifying two promoters and one transgene that would result in target levels of pro-vitamin A elevation without any obvious phenotypic abnormalities.

**Supplementary figures**

**Figure S1** Determination of transgene copy number in transgenic Cavendish banana lines by Southern blot analysis. Genomic DNA from each line was digested with the indicated restriction enzyme and hybridized with a transgene specific probe. M, DNA molecular weight marker II (Roche); +, plasmid DNA positive control; WT, wild-type.

**Figure S2** Determination of transgene copy number in transgenic Cavendish banana lines by Southern blot analysis. Genomic DNA from each line was digested with the indicated restriction enzyme and hybridized with a transgene specific probe. M, DNA molecular weight marker II (Roche); +, plasmid DNA positive control; WT, wild-type.

**Figure S3** Representative HPLC chromatogram of the main carotenoids in wild-type and transgenic Cavendish banana. HPLC chromatograms of carotenoid extracts from (a) wild-type Cavendish banana and (b) Ubi-*MtPsy2a* transgenic line FT294.

**Figure S4** Transgene expression analysis in selected *MtPsy2a* transgenic Cavendish banana lines by reverse transcriptase-PCR (RT-PCR). (a) DNase-treated total RNA templates and (b) cDNA templates. WT, wild-type with WT1= FT167 and WT2= FT430; NTC, non-template control; G+, genomic DNA positive control; P+, plasmid DNA positive control and M, HyperLadder I (Bioline) marker.

**Figure S5** Transgene expression analysis in selected *ZmPsy1* transgenic Cavendish banana lines by reverse transcriptase-PCR (RT-PCR). (a) DNase-treated total RNA templates and (b) cDNA templates. WT, wild-type with WT1= FT167 and WT2= FT430; NTC, non-template control; G+, genomic DNA positive control; P+, plasmid DNA positive control and M, HyperLadder I (Bioline) marker.

**
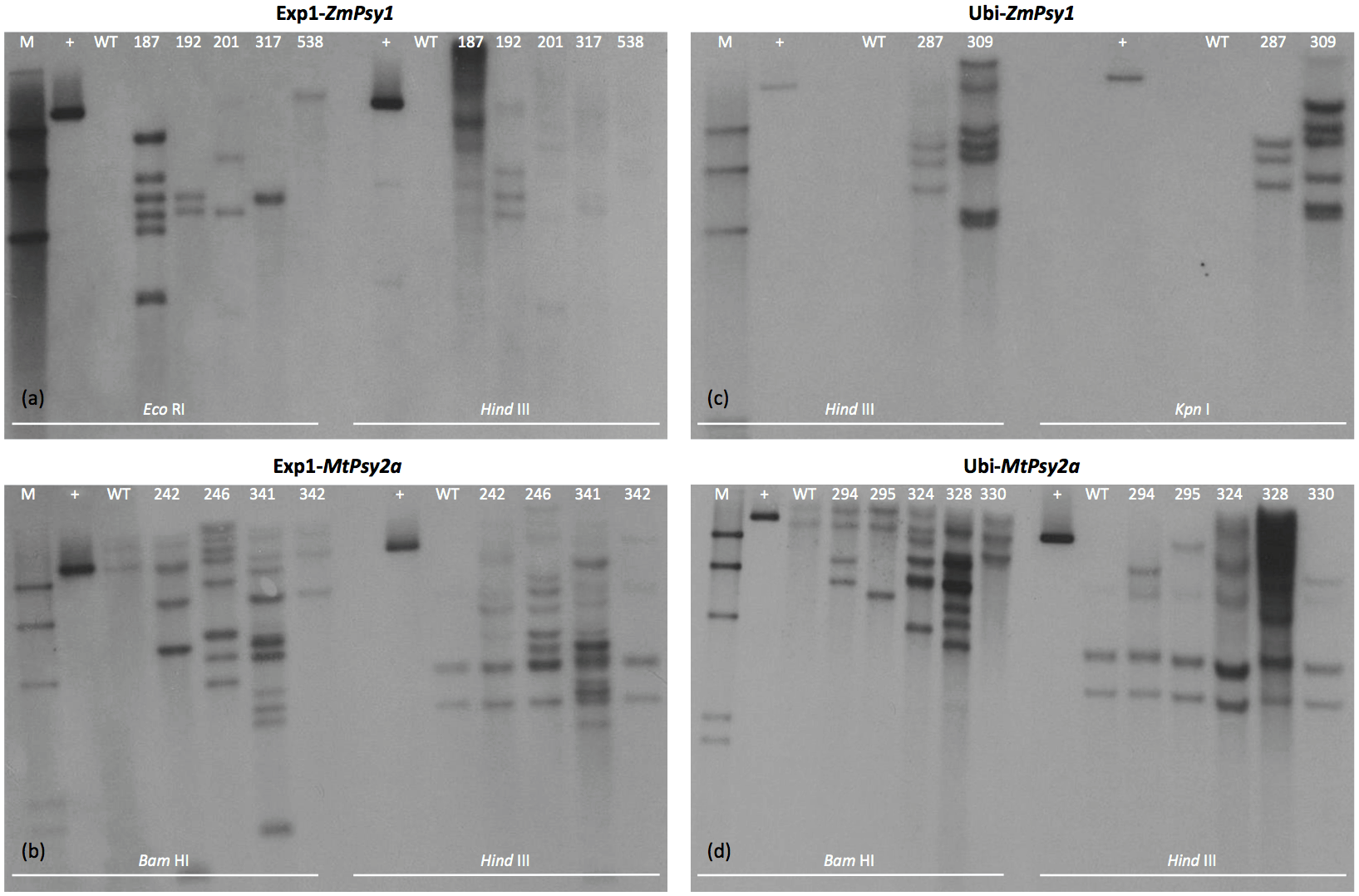
**

**Figure S1** Determination of transgene copy number in transgenic Cavendish banana lines by Southern blot analysis. Genomic DNA from each line was digested with the indicated restriction enzyme and hybridized with a transgene specific probe. M, DNA molecular weight marker II (Roche); +, plasmid DNA positive control; WT, wild-type.

**
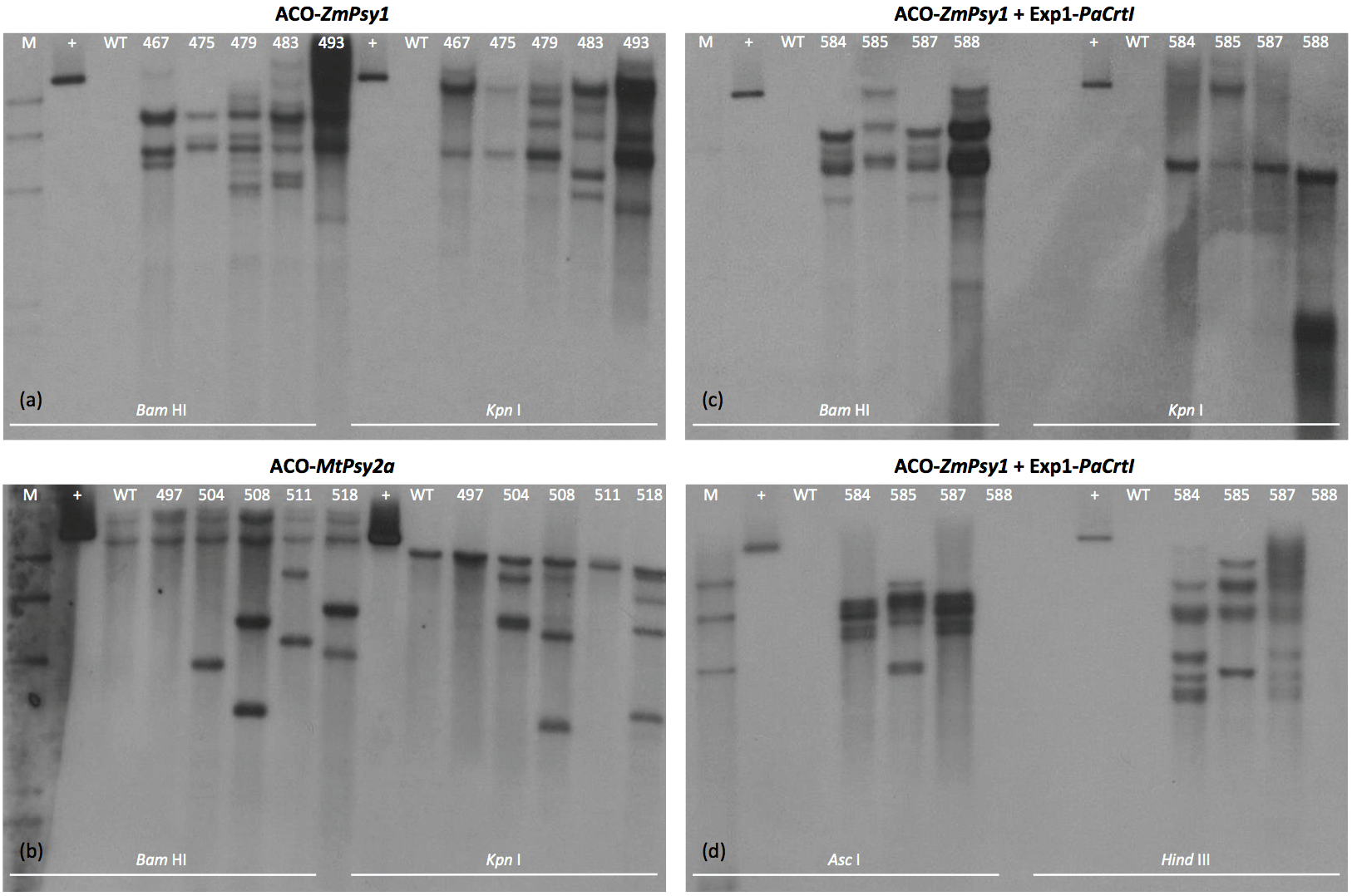
**

**Figure S2** Determination of transgene copy number in transgenic Cavendish banana lines by Southern blot analysis. Genomic DNA from each line was digested with the indicated restriction enzyme and hybridized with a transgene specific probe. M, DNA molecular weight marker II (Roche); +, plasmid DNA positive control; WT, wild-type.

**
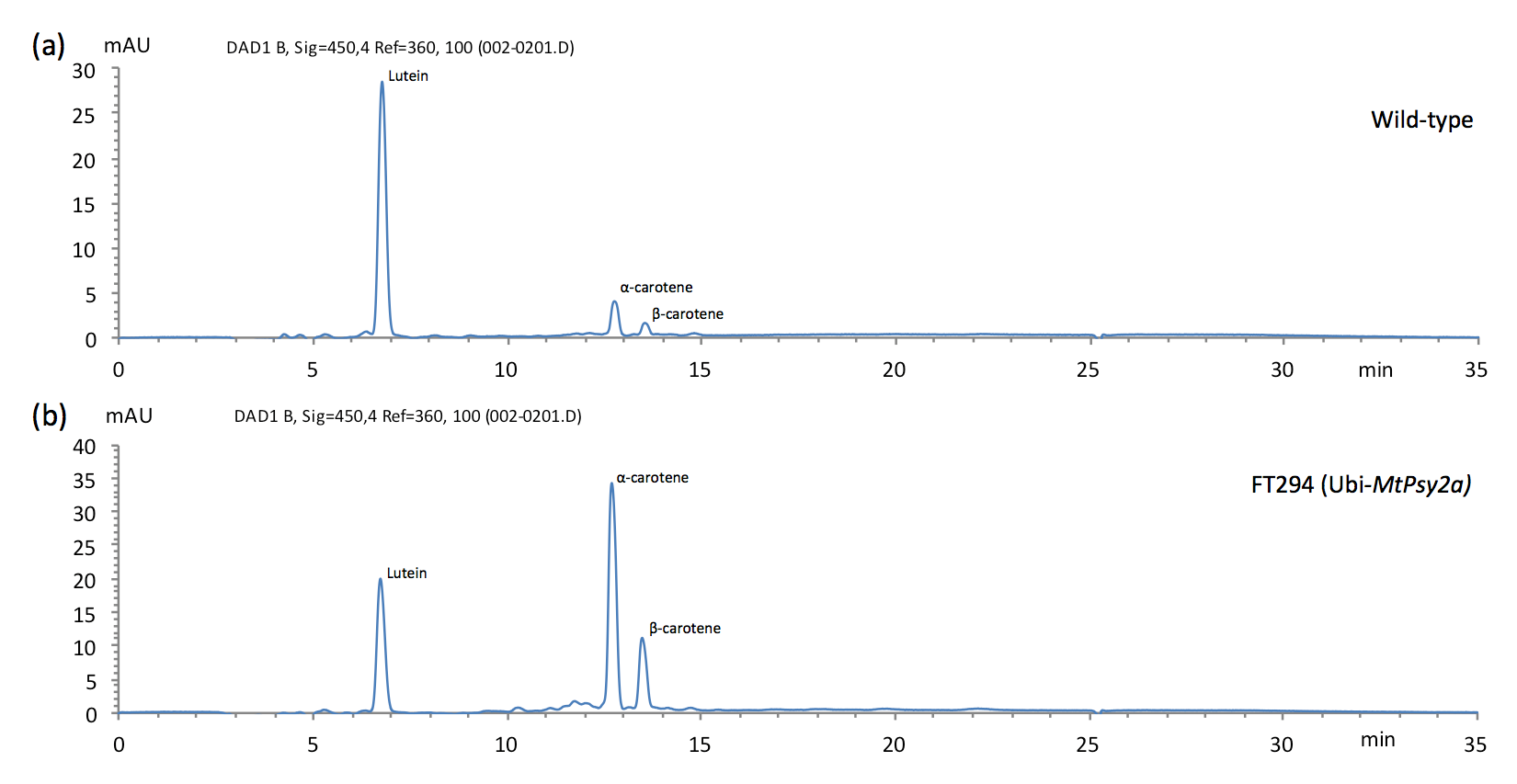
**

**Figure S3** Representative HPLC chromatogram of the main carotenoids in wild-type and transgenic Cavendish banana. HPLC chromatograms of carotenoid extracts from (a) wild-type Cavendish banana and (b) Ubi-*MtPsy2a* transgenic line FT294.

**
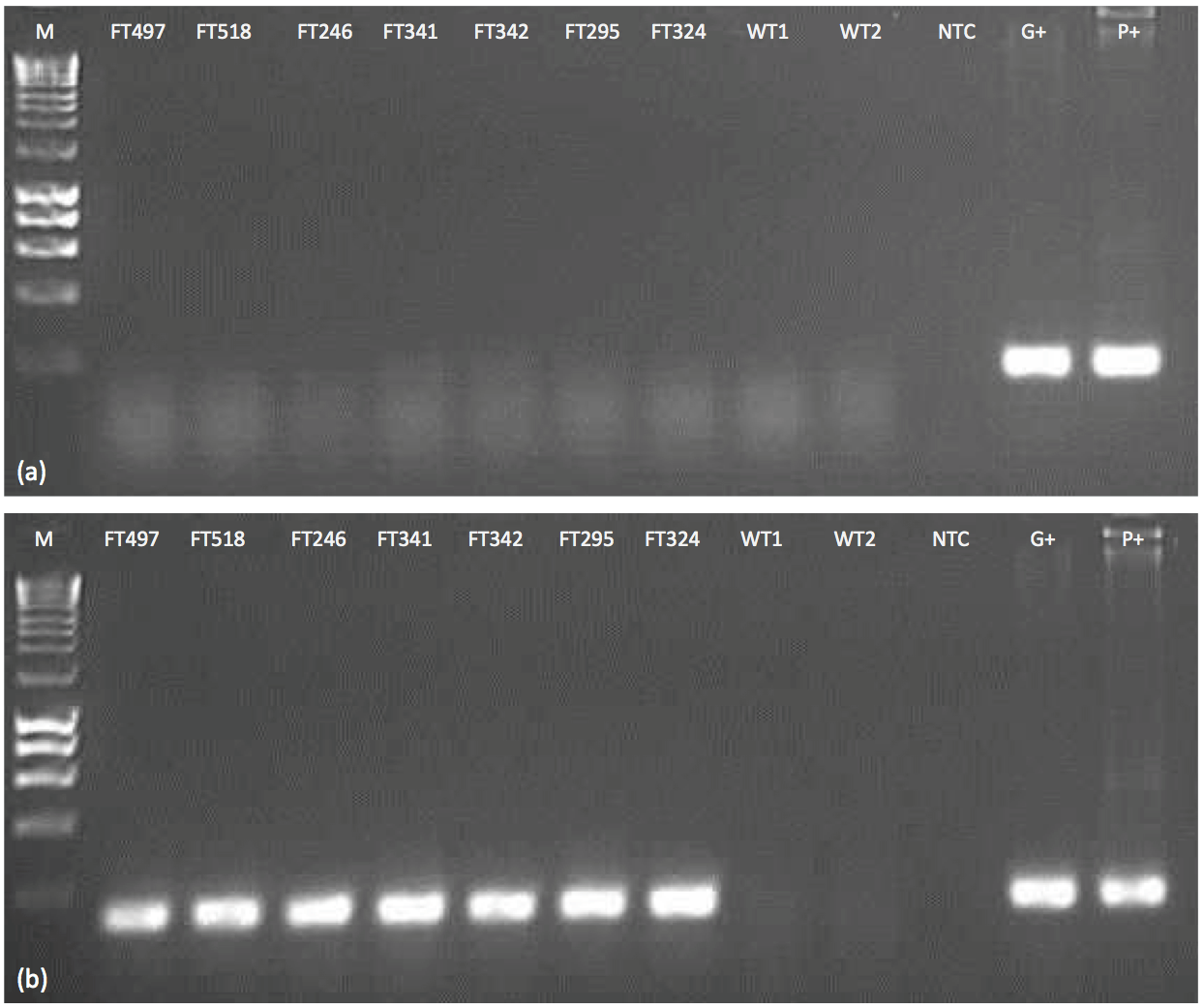
**

**Figure S4** Transgene expression analysis in selected *MtPsy2a* transgenic Cavendish banana lines by reverse transcriptase-PCR (RT-PCR). (a) DNase-treated total RNA templates and (b) cDNA templates. WT, wild-type with WT1= FT167 and WT2= FT430; NTC, non-template control; G+, genomic DNA positive control; P+, plasmid DNA positive control and M, HyperLadder I (Bioline) marker.

**
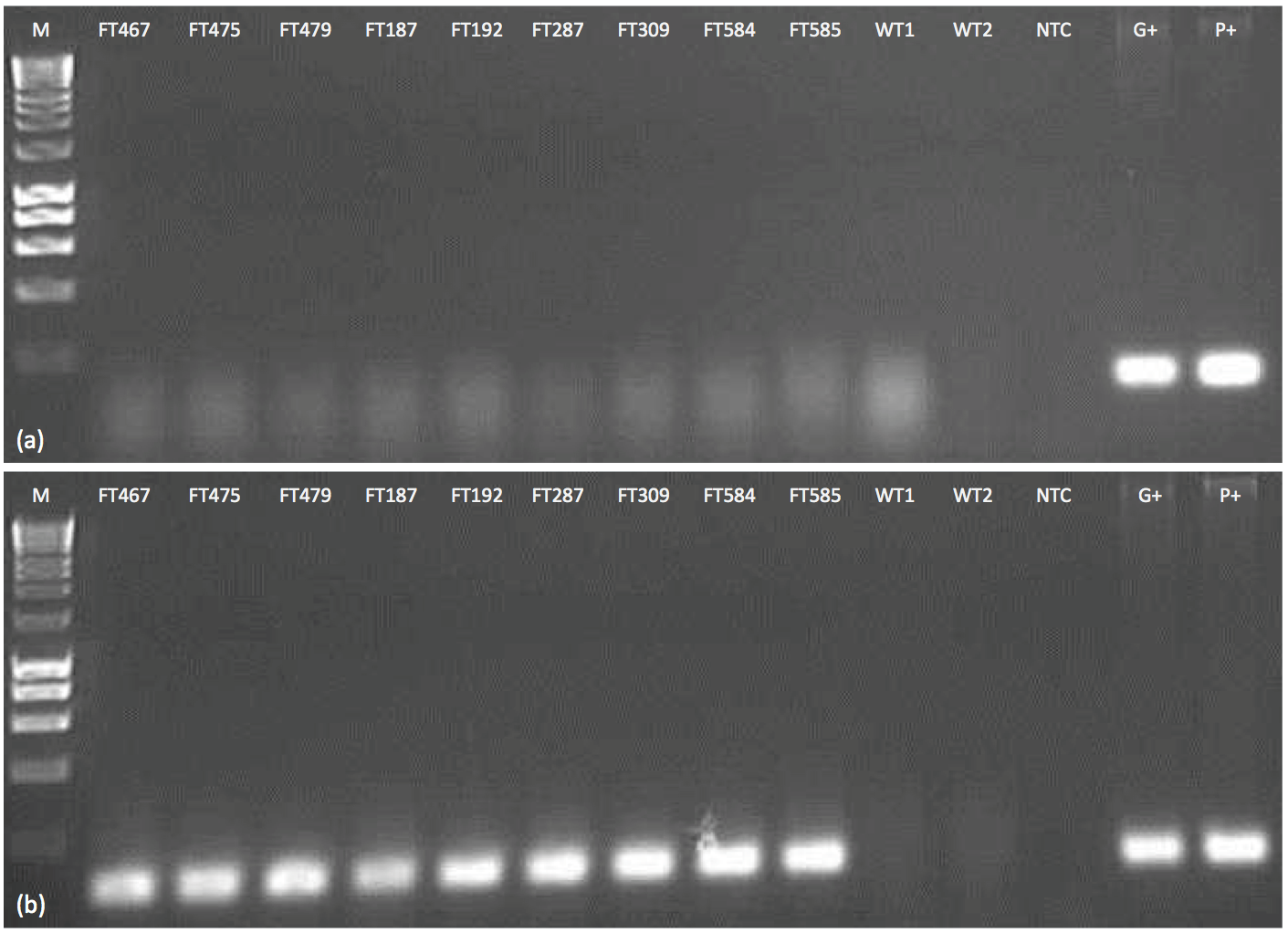
**

**Figure S5** Transgene expression analysis in selected *ZmPsy1* transgenic Cavendish banana lines by reverse transcriptase-PCR (RT-PCR). (a) DNase-treated total RNA templates and (b) cDNA templates. WT, wild-type with WT1= FT167 and WT2= FT430; NTC, non-template control; G+, genomic DNA positive control; P+, plasmid DNA positive control and M, HyperLadder I (Bioline) marker.

**Supplementary table**

**Table S1** List of oligonucleotide primer sequences used for RT-PCR and qRT-PCR

| **Target sequence** | **Primer** | **Sequence (5’ 🡪 3')** | **Amplicon size (bp)** |
| --- | --- | --- | --- |
| *CYP* | qCYP F | GACGGTTCACGCCTCTGTG | 97 |
|  | qCYP R | TGGCTCCTGCTGACGATAATG |  |
| *RPS2* | qRPS2-F | ACTCAACCGTCTTCCCAAAAG | 110 |
|  | qRPS2-R | TCACAATATCAGGCAATCCCG |  |
| *MtPsy2a* | qAPsy2a F | CCAAGGCAGTAATCGTAGGC | 155 |
|  | qNos R | ATGTGATAATCATCGCAAGACC |  |
| *ZmPsy1* | qZmPsy1 F3 | CATTGAGAAATGGCCAGACC | 104 |
|  | qNosPsy1 R3 | AAGACCGGCAACAGGATTC |  |
| *PaCrtI* | qCrtI F2 | CAAAAGCGACAGCAGGTTTG | 159 |
|  | qNos R | ATGTGATAATCATCGCAAGACC |  |

**Discussion related to relative proportions of PVA carotenoids in banana**

The vitamin A nutritional value of a crop is not only dependent on the total amount of PVA carotenoids present but also dependent on the relative proportion of each of them. With twice the conversion rate to retinol than α-carotene, β-cryptoxanthin and other PVA carotenoids, β-carotene is the best PVA carotenoid for biofortification. Unfortunately, Cavendish banana, but also EAHB naturally accumulate significantly more α-carotene than β-carotene and undetectable levels of β-cryptoxanthin. In wild-type Cavendish, close to 72 % of PVA carotenoids consists of α-carotene and, irrespective of the transgene used, more than 70% of PVA carotenoids in all transgenic plants analysed also consists of α-carotene. In contrast, Golden Rice 2 was reported to have more than 83% of its total carotenoid consisting of β-carotene alone (Paine *et al.,* 2005). A simple explanation for the important proportional accumulation of α-carotene in banana could be a preference of the carotenoid metabolic flux towards the ε,β-carotene branch, probably in conjunction with a metabolic bottleneck at the α-carotene hydroxylases stage resulting in a slow down-stream processing of carotenes to lutein. Importantly, increasing the amount of phytoene synthase does not appear to impact the way carotenes are processed through the metabolic pathway in banana.
